# A Lesion in the Corpus Callosum due to Eosinophilic Meningitis Caused by *Angiostrongylus cantonensis*

**DOI:** 10.4269/ajtmh.18-0808

**Published:** 2019-06

**Authors:** Verajit Chotmongkol, Sittichai Khamsai

**Affiliations:** Department of Medicine, Faculty of Medicine, Khon Kaen University, Khon Kaen, Thailand

**Figure 1.**
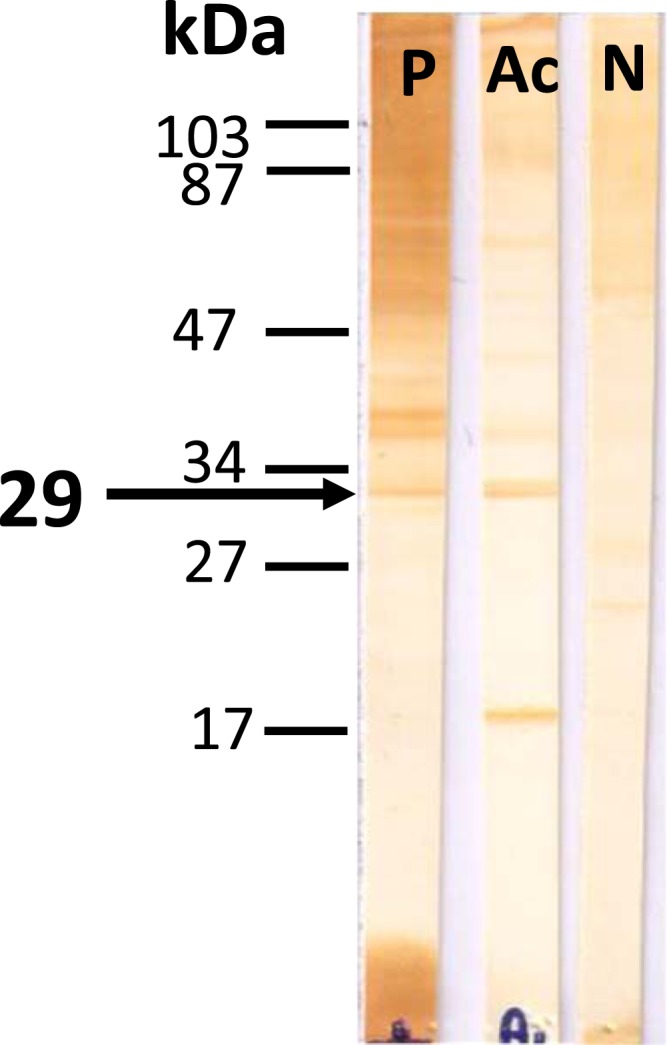
The representative of immunoblot patterns showing antibody reactivity to the *Angiostrongylus cantonensis* somatic antigen. Blots developed with pooled positive reference sera (P), pooled negative reference sera (N), and angiostrongyliasis patient serum (Ac) showing a strongly positive band at 29 kDa. This figure appears in color at www.ajtmh.org.

**Figure 2.**
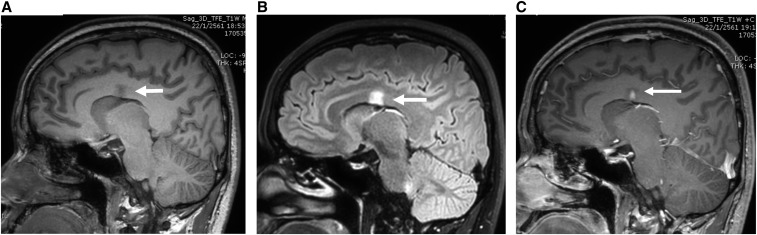
Magnetic resonance imaging of the brain showed a focal lesion at the body of the corpus callosum, which exhibited low signal intensity on sagittal T1-weighted images (**A**), high signal intensity on fluid-attenuated inversion recovery images (**B**), and nodular enhancement after administration of gadolinium (**C**).

**Figure 3.**
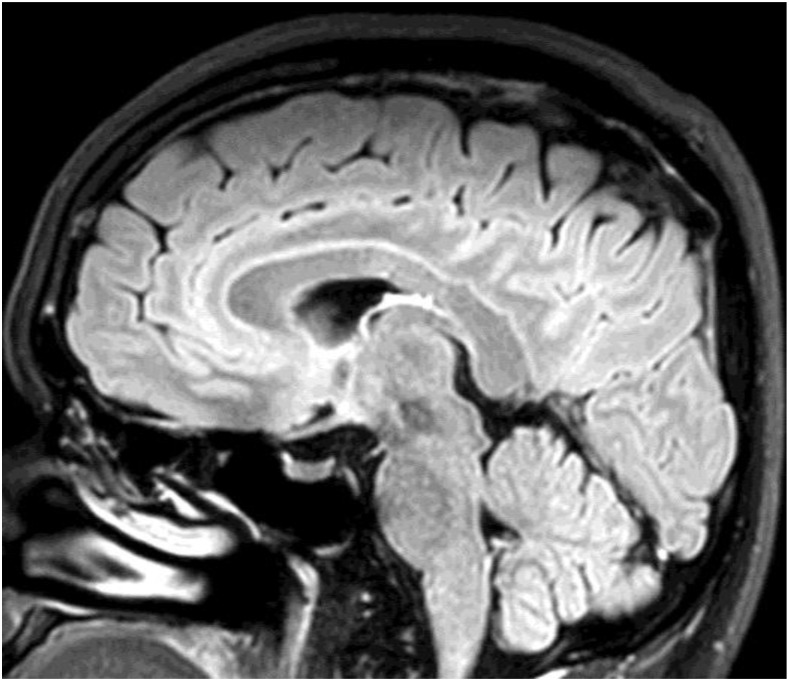
Sagittal fluid-attenuated inversion recovery magnetic resonance imaging of the brain at a 6-month follow-up showing complete resolution of the lesion.

The etiologies of diseases involving the corpus callosum include congenital factors, demyelinating, infection, trauma, neoplasm, and ischemia.^1^ We report a case of eosinophilic meningitis with a lesion at the corpus callosum caused by *Angiostrongylus cantonensis*. A previously healthy 38-year-old man presented with a severe headache that had lasted for 3 days. The headache had a continuous pressing quality and bilateral involvement. One month earlier, he had eaten raw pila snail and freshwater shrimp. He was afebrile, and no nuchal rigidity was detected. The findings of other neurological examination were unremarkable. A blood test revealed a white blood cell (WBC) count of 11,150 cells/mm^3^ with 21% eosinophils. Computed tomography of the brain without contrast administration suggested generalized brain swelling. A lumbar puncture revealed an opening pressure of 250 mm H_2_O. Cerebrospinal fluid (CSF) analysis showed a WBC count of 368 cells/mm^3^ with 60% eosinophils, a protein level of 156 mg/dL, and a glucose level of 44 mg/dL (concurrently a blood glucose level of 125 mg/dL). The results of Gram staining, India ink preparation, and culture for bacteria were negative. Blood and CSF serological study for angiostrongyliasis using immunoblotting were positive for the 29-kDa antigenic band (Figure 1).^2^ He was treated with a 2-week course of prednisolone and supportive treatment,^3^ and the headache improved. However, at a follow-up 2 weeks later, he complained of numbness of the left side of his face and the left upper limb. Magnetic resonance imaging of the brain showed a focal lesion at the body of corpus callosum, which exhibited low signal intensity on T1-weighted images, high signal intensity on T2-weighted images and fluid-attenuated inversion recovery images, and nodular enhancement after administration of gadolinium (Figure 2). Other similar lesions were found at the right parieto-occipital region and the left lentiform nucleus. The brain lesions were completely resolved at a 6-month follow-up, and the numbness disappeared with time (Figure 3).
